# Determination of the band parameters of bulk 2H-MX_2_ (M = Mo, W; X = S, Se) by angle-resolved photoemission spectroscopy

**DOI:** 10.1038/srep36389

**Published:** 2016-11-02

**Authors:** Beom Seo Kim, Jun-Won Rhim, Beomyoung Kim, Changyoung Kim, Seung Ryong Park

**Affiliations:** 1 Center for Correlated Electron Systems, Institute for Basic Science, Seoul, 08826, Korea; 2 Department of Physics and Astronomy, Seoul National University, Seoul, 08826, Korea; 3 Department of Physics, Incheon National University, Incheon, 22012, Korea; 4 Max-Planck-Institut für Physik komplexer System, 01187, Dresden, Germany; 5 Department of Physics, Pohang University of Science and Technology, Pohang, 37673, Korea; 6 Advanced Light Source, Lawrence Berkeley National Laboratory, Berkeley, CA 94720, USA

## Abstract

Monolayer MX_2_ (M = Mo, W; X = S, Se) has recently been drawn much attention due to their application possibility as well as the novel valley physics. On the other hand, it is also important to understand the electronic structures of bulk MX_2_ for material applications since it is very challenging to grow large size uniform and sustainable monolayer MX_2_. We performed angle-resolved photoemission spectroscopy and tight binding calculations to investigate the electronic structures of bulk 2H-MX_2_. We could extract all the important electronic band parameters for bulk 2H-MX_2_, including the band gap, direct band gap size at K (-K) point and spin splitting size. Upon comparing the parameters for bulk 2H-MX_2_ (our work) with mono- and multi-layer MX_2_ (published), we found that stacked layers, substrates for thin films, and carrier concentration significantly affect the parameters, especially the band gap size. The origin of such effect is discussed in terms of the screening effect.

The successful exfoliation of graphene^1^,^2^,^3^ is important on its own right but also has triggered the intensive/extensive research on similar two-dimensional layered materials^4^,^5^. Transition metal dichalcogenides (TMDs) such as NbSe_2_ and MoS_2_ have strong in-plane covalent and weak out-of-plane van der Waals bonds. Such bonding character reduces the dimensionality from 3D to 2D and allows us to obtain monolayer systems by the exfoliation method. Monolayer TMDs often exhibit qualitatively different electronic properties compared to the bulk^6^,^7^,^8^.

Among the TMDs, the group 6 TMDs, MX_2_ (M = Mo, W; X = S, Se), exhibit interesting electronic properties such as indirect (bulk) to direct (monolayer) band gap transition^6^,^7^, valley degeneracy^9^ and spin-orbit interaction (SOI) induced spin band splitting at the K and -K points of the hexagonal Brillouin zone^10^. Exploiting these fundamental electronic properties, the valley degeneracy could be lifted by using circularly polarized light^11^,^12^,^13^,^14^,^15^ and valley Hall effect was observed in monolayer MX_2_^16^,^17^,^18^. These raised the notion of the valleytronics^19^,^20^,^21^,^22^,^23^,^24^,^25^.

These low energy electronic properties of monolayer MX_2_ are found to be explained within a minimal model, the so-called massive Dirac fermion model^9^. The model has only three independent parameters: the effective hopping (*t*), band gap without SOI (*Δ*), and spin band splitting (2λ). The details of the model are described in section 2.1. The electronic structure of monolayer MX_2_ has been measured by angle-resolved photoemission spectroscopy (ARPES), which has confirmed the direct band gap and the spin band splitting at the K and -K points^7^,^26^,^27^,^28^,^29^,^30^,^31^. More importantly, band parameters could be extracted from the ARPES data^7^,^26^,^27^,^28^,^29^,^30^,^31^,^32^,^33^,^34^. The extracted values of *Δ* and 2*λ* are 1.465 and 0.15 eV for the epitaxial monolayer MoS_2_ on Au(111), and 1.67 and 0.18 eV for monolayer MoSe_2_ grown on bilayer graphene^7^,^29^. 2*λ* of monolayer WS_2_ grown on Au(111) has been recently measured and found to be 0.42 eV^30^. These results show that the massive Dirac fermion parameters for monolayer MX_2_ can be affected not only by the chemical composition but also by other factors such as the substrate and the carrier concentration of the system^29^,^30^,^31^,^34^.

For the MX_2_ based electronic devices, it is natural to start with multi-layer MX_2_ films which are closer to bulk rather than monolayer since it is difficult to grow high-quality monolayer-MX_2_ in wafer scale. Then, the information on the electronic structure of bulk MX_2_ is also important as stacked layers affect the electronic structure. Moreover, it is interesting to see how the electronic structure of monolayer MX_2_ evolves as it is stacked into the bulk, and also to understand how the massive Dirac fermion model connects to the bulk electronic structure. 2H-MX_2_ is the most abundant bulk form of MX_2_ in which in-plane polarization of the MX_2_ monolayers are antiparallel to that of the nearest neighbor layers, resulting in unit-cell doubling. Previous ARPES studies have shown that the valence band maximum (VBM) is located at the in-plane Γ-point in various bulk 2H-MX^32^,^33^,^34^. As a result, these materials have an indirect band gap^7^,^33^. Spin band splitting has also been observed^31^. However, it has not been systematically studied in regards to the material dependent band-gap, including the direct band gap at the in-plane K point in bulk 2H-MX_2_ (M = Mo, W; X = S, Se). For example, the direct band gap at the in-plane K point has been investigated only for 2H-WSe_2_^34^. It is therefore desired to systematically investigate electronic structures of 2H-MX_2_.

We performed ARPES experiments to investigate all the important electronic band parameters of bulk 2H-MoS_2_, MoSe_2_, WS_2_ and WSe_2_ including band gap, direct band gap at K-point and spin splitting. We also carried out tight binding calculations to interpret our ARPES data and to provide a simple understanding of electronic structure evolution from monolayer to bulk 2H-MX_2_. We could successfully extract all the parameters of bulk 2H-MX_2_ from the ARPES data. Upon comparing the parameters of bulk 2H-MX_2_ with those of previously studied MX_2_ thin films, including monolayer, we found that the direct band gap at the K point is significantly affected by the number of layers and doped electron density, while other parameters such as spin splitting size does not change appreciably. We will discuss the underlying physics behind the behavior of the parameters.

## Results and Discussions

### Tight binding calculations for electronic structure evolution from monolayer to bulk 2H-MX_2_

**Figure 1:**
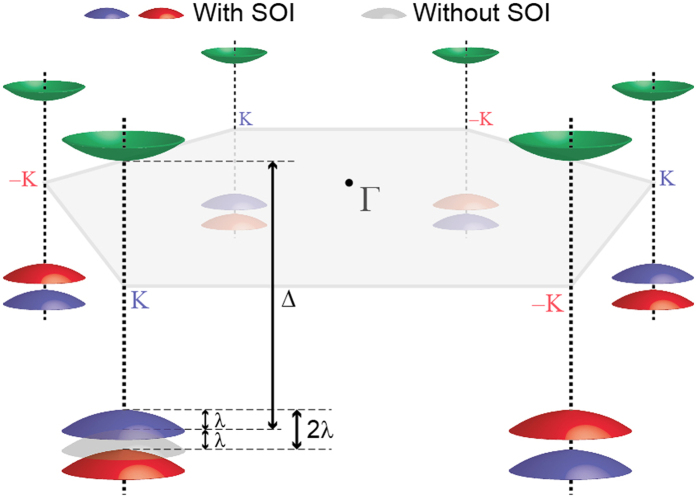
Schematic sketch of the massive Dirac fermion model. Gray VB at the front-left K point is for the case without SOI while red/blue VB edges correspond to the spin up/down states for finite SOI.

Low energy electronic structure of monolayer MX_2_ is well described by the massive Dirac fermion model. We will try to show that the band dispersion of bulk MX_2_ near the in-plane K point can be also described within the model. Note that while the inversion symmetry is restored in the bulk and thus the valley physics is removed, the characteristics of the monolayer such as the spin band splitting remains in the dispersion at the K point. Figure 1 is a schematic sketch of the massive Dirac fermion model. Two cases are illustrated in the figure, one without SOI and the other with SOI. The Hamiltonian of the massive Dirac fermion model including SOI reads


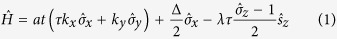


where *a* is the lattice constant, *t* the effective hopping parameter, *τ* the valley index, 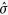 the Pauli matrices for the basis functions, *Δ* the direct band gap size without SOI, 2*λ* the SOI induced spin band splitting size, and 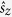 the Pauli matrix for spin (see ref. 9 for more details). Note that there are only three free parameters in this model, *Δ*, 2*λ*, and *t*. We performed tight binding calculations with a focus on how the electronic structure at the in-plane K and Γ points evolves from monolayer MX_2_ to bulk MX_2_. Our calculations show that band dispersion along *k*_*z*_ at the in-plane K point is zero and can still be described by the band dispersion of the massive Dirac fermion parameters, whereas band dispersion along *k*_*z*_ at the in-plane Γ point is strong enough to induce direct to indirect band-gap transition. As noted above, only the dispersion of the model can be used for the 2H-MX_2_, since the broken inversion symmetry of monolayer is recovered in bulk 2H-MX_2_. Spin states are, for instance, degenerate in bulk 2H-MX_2_.

The evolution of the dispersion relations from the monolayer to the bulk system at the Γ and K point are studied by investigating how the eigenstates in different layers become mixed together as a result of the stacking. Details are in Supplementary Material (SM) and only the main results are given. Here, we neglect the SOI which does not affect the *k*_*z*_ dependences of the energy spectra due to its on-site character. Then, we obtain the energy spectra along *k*_*z*_ as


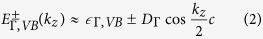


at the in-plane Γ point, and


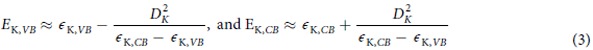


at the in-plane K point. The subscripts VB and CB represent the valence and conduction bands, respectively. *∈*_Γ*,VB*_ and *∈*_K*,VB*_ are energies at Γ and K point for the corresponding bands of monolayer MX_2_. Constants *D*_Γ_ and *D*_K_ are described in SM. One can note that the width of this VB at the in-plane Γ point is 2*D*_Γ_ which is evaluated to be approximately 0.86 eV for MoS_2_ from the tight binding parameters and the lattice constants in refs 35, 36, 37. This is comparable with the experimental result.

As shown in the above results, two high symmetry points Γ and K of the monolayer MX_2_ show completely different responses to the stacking. The VB at the Γ point gains strong dispersions along *k*_*z*_ while the VB and CB at the K point are almost dispersionless and experience only small shifts (*D*_*K*_ ≈ 0.0263 eV). This distinction originates from the difference in the orbital compositions between them and the three-fold rotational symmetry of the system.

At the Γ point, the eigenstates mainly consist of the out-of-plane orbitals such as 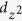 orbitals at M atoms and *p*_z_ orbitals at X atoms. As a result, the overlap integrals between them in different layers are expected to be large compared to the in-plane orbitals. Also, it is impossible to have the phase cancellation related to the factor 
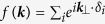
 at the Γ point (***k***_**⊥**_ = 0) so that there is no chance to remove the dispersion along *k*_*z*_ direction. This is why we have strong dispersions in the VB along *k*_*z*_ at the in-plane Γ point. On the other hand, the eigenstates on the CB at the in-plane Γ point consist of *p*_*x*_ and *p*_*y*_ orbitals at X atoms and their dispersions along *k*_*z*_ direction induced by stacking are relatively weak.

At the K point, on the contrary, both the conduction and valence electrons only have the in-plane orbital components (*p*_*x*_ and *p*_*y*_) in X atoms. Although there are out-of-plane 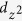 orbitals in M atoms, they give next order terms when layers are stacked since the M-M or M-X distances between neighboring layers are quite far compared to the X-X distance. This in-plane character of the constituent orbitals immediately makes us to expect smaller dispersions for the VB and CB along *k*_*z*_ direction at the in-plane K point than that of the VB at the in-plane Γ point. However, we have shown that even these small dispersions are suppressed and the band spectra along *k*_*z*_ direction becomes almost flat due to the graphene-like phase cancellation among the nearest neighboring hopping processes stemming from the C_3_ symmetry of the system^38^.

### ARPES measurements on bulk 2H-MX_2_

**Figure 2:**
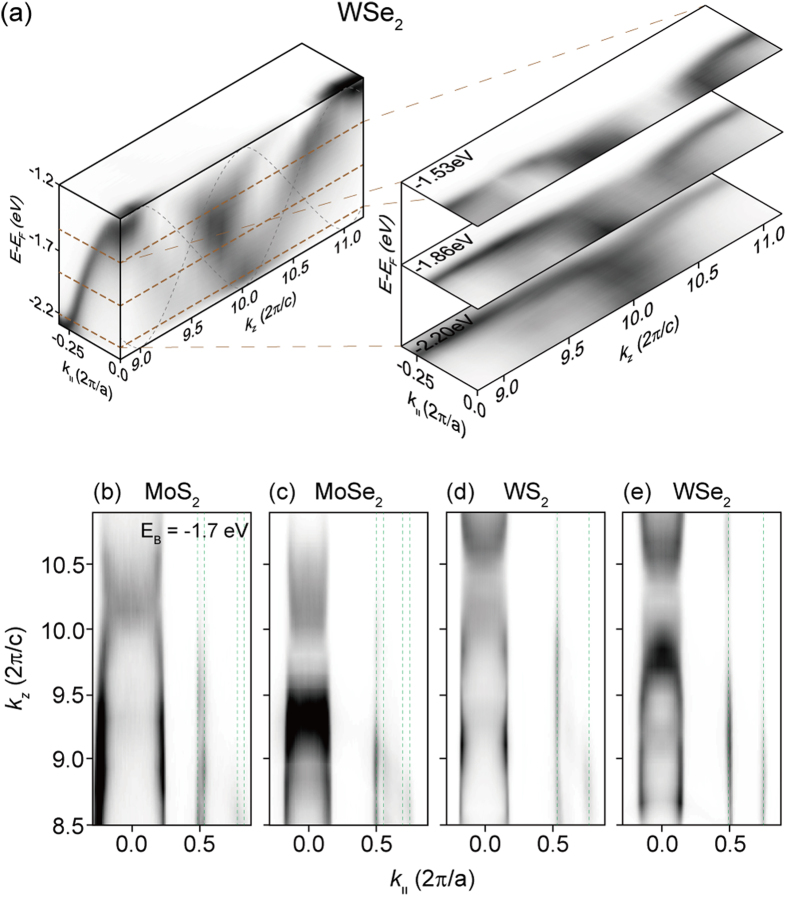
Photon energy dependent ARPES data. (**a**) Intensity plot of WSe_2_ ARPES data in energy and momentum (*k*_*z*_, *k*_||_) space. *k*_*z*_ dependent ARPES is taken by using different photon energies. *k*_*z*_ of 9.0 and 11.0 correspond to the incident photon energies of 58 and 94 eV, respectively. The black dashed lines indicate the expected *k*_*z*_ dispersion of the bands with *D*_Γ_ = 0.5 eV [Eq. (2)]. Three selected cuts on the right hand side along the brown dashed lines are ARPES intensity maps at constant energies in the momentum space (*k*_*z*_, *k*_||_). Also shown are ARPES intensity maps of (**b**) MoS_2_, (**c**) MoSe_2_, (d) WS_2_, (**e**) WSe_2_ at a constant binding energy of −1.7 eV. The dashed lines are guides to eye for the electronic states near the K point. These lines are straight along the *k*_*z*_.

We first performed photon-energy dependent ARPES to obtain the *k*_*z*_ dispersion of the electronic band. Figure 2(a) shows the ARPES data taken with incident photon energies between 50 and 100 eV near the in-plane Γ point. Black dashed lines indicate band dispersions expected from Eq. (2). The data is in good agreement with the calculation results and show strong *k*_*z*_ dispersions. The breadth in the ARPES data in the energy direction is due to the finite escape depth of the ARPES process (finite *k*_*z*_ resolution). *k*_*z*_ dispersions in MoS_2_, MoSe_2_, and WS_2_ near the in-plane Γ point are as strong as that in WSe_2_ [Fig. 2(b–d)].

**Figure 3:**
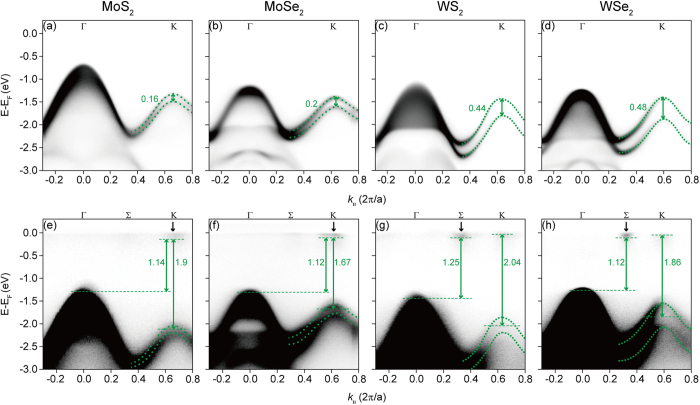
Electronic structure before and after K evaporation. (**a**–**d**) ARPES data along the Γ to K from pristine MoS_2_, MoSe_2_, WS_2_, and WSe_2_. Green dotted lines indicate the band dispersions near the K point. (**e**–**h**) ARPES data after potassium evaporation. The concentration of the doped electrons by potassium evaporation can be estimated from the Fermi surface volume. The estimated electron doping concentrations are 1.7 × 10^13^ cm^−2^, 2.5 × 10^13^ cm^−2^, 3.5 × 10^13^ cm^−2^, and 2.6 × 10^13^ cm^−2^ for MoS_2_, MoSe_2_, WS_2_, and WSe_2_, respectively.

On the other hand, photon-energy dependent ARPES data show no *k*_*z*_ dispersion near the in-plane K point as seen in Fig. 2(b–e), consistent with our calculation results in Eq. (3). Dashed lines in Fig. 2(b–e) are guides to eye and are straight (that is, no *k*_*z*_ dispersion). Since the energy of the band at a specific in-plane momentum is the same regardless of *k*_*z*_, ARPES spectra near the K point are very sharp in comparison to the Γ point data, both in the energy and in-plane momentum directions. This fact can be seen in Fig. 2(b–e) as well as in Fig. 3(a–d).

In order to extract the electronic band parameters, we need ARPES data along the in-plane Γ to K direction (see Fig. 3). 2*λ* of MoS_2_, MoSe_2_, WS_2_, and WSe_2_ can be clearly observed in the data shown in Fig. 3(a–d). 2λ is drastically increased as the transition metal changes from Mo to W since 2*λ* mostly relies on the atomic spin-orbit coupling of the transition metal atom.

In order to observe the direct band gap at the K point and the indirect band gap, it is necessary to see the bottom of the CB. The problem is that the states are not occupied and thus cannot be observed by ARPES. One way to circumvent the problem is to populate the CB bottom by potassium (K) dosing^7^,^29^,^31^. K has very low electron affinity and, when dosed on the sample surface, provides electrons. ARPES experiments after K evaporation reveal the conduction band minimum (CBM) from which we can determine *Δ* [Fig. 3(e–h)]. The energy of the CBM is determined from the onset of the photoemission intensity, as indicated by dashed lines near the Fermi energy at the K point for MoS_2_ and MoSe_2_ [Fig. 3(e,f)] and at the Σ point for WS_2_ and WSe_2_ [Fig. 3(g,h)]. A local CBM for the K point for WS_2_ and WSe_2_ is also observed as indicated by dashed lines. The CBM is found to be located at the K point in MoS_2_ and MoSe_2_, while it is located at the Σ point in WS_2_ and WSe_2_. We note that the CBM of monolayer WS_2_ and WSe_2_ is located at the Σ point instead of K point. This is because the *k*_*z*_ dispersion at the Σ point for WS_2_ and WSe_2_ causes the CBM at the Σ point to be located even lower than that at the K point.

The effective hopping integral, *t*, can also be estimated by fitting the band dispersion with the band dispersion of the massive Dirac fermion model. *t* is linearly proportional to the slope of band dispersion at off K point, which is for example *k*_||_ = 0.5 (2 π/a) in Fig. 3. Therefore, *t* in WS_2_ and WSe_2_ is clearly greater than that in MoS_2_ and MoSe_2_, and so is the mobility when electrons or holes are doped into these systems. The extracted *t* values for MoS_2_, MoSe_2_, WS_2_, and WSe_2_ are given in Table 1. Here, we assume that the CB dispersion which cannot be measured is mirror-symmetric with the VB dispersion. This is not an unreasonable assumption considering the band calculation results^36^.

All the parameters of 2H-MX_2_ (this work) and the known parameters of mono- and multi-layer MX_2_ are summarized in Table 1. We show in the first column the doped electron density by potassium dosing since doped electron density can affect some of parameters, especially *Δ*^34^. In the second through fourth columns, the three fundamental parameters of the model are summarized. In the last two columns, other interesting parameters, which are direct band gap at K point (*Δ*–*λ*) and indirect band gap, are also summarized. Comparing the fundamental parameters of 2H-MX_2_ and monolayer MX_2_, we notice that spin band splitting (2*λ*) is about 20 meV larger in 2H-MX_2_. This is consistent with the results of optical experiments^39^. We also find that the doped electron density does not affect the size of the spin band splitting (Fig. 3).

On the other hand, the story for *Δ* is different from that of the spin band splitting (2*λ*). Unlike the spin band splitting, *Δ* is affected by various factors such as the density of doped electrons. In order to measure *Δ* or direct band gap (*Δ*–*λ*) by ARPES, it is necessary to introduce electrons into MX_2_ to populate the CBM. The measured value of *Δ*–*λ* by ARPES in such a way is clearly smaller than that measured by STM on undoped MX_2_ even though there is some variation in the reported STM values^40^,^41^,^42^. The observed trend is attributed to the fact that the doped electrons enhance the screening and thus reduce the size of the direct band gap^7^,^40^,^43^. Likewise, it is expected that stacked layers or metallic substrates for thin film play a similar role in the screening effect and thus affect the band gap size. The effects on the band gap reduction from stacked layers and bilayer graphene substrate appear to be similar since the band gaps for 2H-MoSe_2_ and 1ML MoSe_2_/bilayer graphene measured by ARPES are almost the same^7^. On the other hand, the effect from Au substrate must be much larger, considering the fact that 1ML MoS_2_/Au(111) has significantly reduced band gap compared to 2H-MoS_2_^29^. However, we note that quantitative estimation of the band gap reduction from stacking or metallic substrates is not possible without the band gap size of a free-standing MX_2_ monolayer.

## Conclusions

In the theoretical part, we found that the band dispersion along the *k*_*z*_ direction at the in-plane K and -K points vanishes in bulk 2H-MX_2_ due to the graphene-like phase cancellation. Therefore, the electronic band dispersions near the in-plane K and -K points in bulk 2H-MX_2_ are well described by the massive Dirac fermion model. In the experimental part, we confirmed the vanishing *k*_*z*_ dispersion at the in-plane K and -K points in bulk 2H-MoS_2_, 2H-MoSe_2_, 2H-WS_2_ and 2H-WSe_2_. All the fundamental band parameters could be extracted for bulk 2H-MoS_2_, 2H-MoSe_2_, 2H-WS_2_ and 2H-WSe_2_. Most importantly, the direct band gap at the K point (*Δ*–*λ*) shows significant variation depending on the doped electron density, the number of stacking layers and the substrates. The direct band gap variation can be attributed to reduction of the direct band gap due to the enhanced screening. Our work provides useful information on the electronic band dispersions of 2H-, monolayer and multi-layer MX_2_ and suggests a way to manipulate the band gap of MX_2_.

## Methods

ARPES data were obtained at the beam line 4.0.3.2 (MERLIN) of the Advanced Light Source equipped with a VG-SCIENTA R8000 analyzer. The total energy resolution was better than 20 meV. Four high quality single crystal samples were purchased from 2D Semiconductors and HQGraphene. All the data were taken under 40 K in a base pressure better than 4 × 10^−11^ Torr. For the photon energy dependence, we used the photon energy between 50 and 100 eV. Alkali Metal Dispensers from SAES Getters were used for potassium evaporation experiments and evaporation was conducted *in situ* with the samples at the measurement position.

## Additional Information

**How to cite this article**: Kim, B. S. *et al.* Determination of the band parameters of bulk 2H-MX_2_ (M = Mo, W; X = S, Se) by angle-resolved photoemission spectroscopy. *Sci. Rep.* **6**, 36389; doi: 10.1038/srep36389 (2016).

**Publisher’s note:** Springer Nature remains neutral with regard to jurisdictional claims in published maps and institutional affiliations.

## Supplementary Material

Supplementary Information

## Figures and Tables

**Table 1:** Electron density (n) and parameters for the massive Dirac fermion model determined from the 2H-MX_2_ ARPES data.

	n (cm^−2^)	*Δ*	2*λ*	*t*	*Δ*–*λ*	Indirect band gap
2H-MoS_2_	1.7 × 10^13^	1.90	0.16	1.01	1.82	1.14
1 ML MoS_2_/Au(111) [29]	>0 (ARPES)	1.465	0.15	1.10*	1.39	
1 ML MoS_2_/graphite [41]	0 (STM)				2.15	
1 ML MoS_2_/graphite [42]	0 (STM)				2.40	
2 ML MoS_2_/graphite [42]	0 (STM)					2.10
3 ML MoS_2_/graphite [42]	0 (STM)					1.75
2H-MoSe_2_	2.5 × 10^13^	1.67	0.20	0.90	1.57	1.25
1 ML MoSe_2_/bilayer graphene [7]	>0 (ARPES)	1.67	0.18	0.90*	1.58	
8 ML MoSe_2_/bilayer graphene [7]	>0 (ARPES)					1.41
1 ML MoSe_2_/bilayer graphene [40]	0 (STM)				2.18	
2H-WS_2_	3.5 × 10^13^	2.04	0.44	1.25	1.82	1.25
1 ML WS_2_/Au(111) [30]			0.42			
2H-WSe_2_	2.6 × 10^13^	1.86	0.48	1.13	1.62	1.12
1 ML WSe_2_/bilayer graphene [31]	>0 (ARPES)		0.475		1.40	
1 ML WSe_2_/bilayer graphene [31]	0 (STM)					1.95

Also given in the table are the values from published ARPES and STM data on monolayers grown on various substrates. The parameters are expressed in unit of eV. Note that t values with *mark are obtained by fitting the dispersions of the published data.
