# Supplementary material for: Determination of the band parameters of bulk 2H-MX2 (M = Mo, W; X = S, Se) by angle-resolved photoemission spectroscopy
Source: Sci Rep. 2016 Nov 2;6:36389. doi: 10.1038/srep36389 (PMC5090988; doi:10.1038/srep36389)
Supplement: Supplementary Information [file srep36389-s1.pdf]

Supplementary Material

Determination of the band parameters of bulk 2H-MX_2 (M = Mo, W; X = S, Se) by angle-resolved photoemission spectroscopy

Beom Seo Kim,[1,2,3] Jun-Won Rhim,[4,*] Beomyoung Kim,[5,6] Changyoung Kim,[1,2] and Seung Ryong Park[3,†]

[1]*Center for Correlated Electron Systems, Institute for Basic Science, Seoul 151-747, Korea*
[2]*Department of Physics and Astronomy, Seoul National University, Seoul 151-747, Korea*
[3]*Department of Physics, Incheon National University, Incheon 406-772, Korea*
[4]*Max-Planck-Institut für Physik komplexer Systeme, 01187 Dresden, Germany*
[5]*Department of Physics, Pohang University of Science and Technology, Pohang 790-784, Korea*
[6]*Advanced Light Source, Lawrence Berkeley National Laboratory, Berkeley, CA 94720, USA*

I. k_z DISPERSION AT THE IN-PLANE Γ POINT

In this supplementary material, we derive the effective Hamiltonian of bulk TMDs along $\mathbf{k}_0 + k_z\hat{z}$ where $\mathbf{k}_0 = \mathbf{\Gamma}$ or $\mathbf{K}$ is a high symmetry point of the monolayer MX_2 and investigate its energy spectra. To obtain the effective Hamiltonian, we first construct the 3D bulk Bloch basis from the eigenstates of the MX_2 monolayer at $\mathbf{k}_0$ we are interested in. Then, we evaluate the matrix elements related to the interlayer couplings which would determine the dispersions along k_z.

First, let us consider the evolution of the valence band at Γ point of the monolayer MX_2. The two Bloch basis functions are given by

$$|\Psi^{l(u)}_{\Gamma,VB}(k_z)\rangle = \frac{1}{\sqrt{N}}\sum_n |\psi^{l(u),n}_{\Gamma,VB}\rangle e^{ink_zc} \tag{1.1}$$

where

$$|\psi^{l(u),n}_{\Gamma,VB}\rangle = \tilde{c}_1|d^{l(u),n}_{z^2}\rangle - \frac{c_1}{\sqrt{2}}\left(|p_z^{A,l(u),n}\rangle - |p_z^{B,l(u),n}\rangle\right). \tag{1.2}$$

The value of c_n is presented in Ref. [1]. Here, $l(u)$ represents the lower(upper) layer in the bulk's unit cell. For each MX_2 layer, the M slab is sandwiched between two X slabs which are denoted by A(upper one) and B(lower one). The unit cell's index along z axis is represented by n.

The on-site energy of $|\Psi^{l(u)}_{\Gamma,VB}(k_z)\rangle$ is the same with that of $|\psi^{l(u),n}_{\Gamma,VB}\rangle$ and expressed by $\epsilon_{\Gamma,VB}$. The band splitting induced by the stacking of MX_2 layers at Γ point is evaluated as

$$\begin{aligned}
\Delta_{\Gamma,VB}(k_z) &= \langle\Psi^u_{\Gamma,VB}(k_z)|H'|\Psi^l_{\Gamma,VB}(k_z)\rangle && (1.3)\\
&= \frac{1}{N}\sum_{n,m}\langle\psi^{u,n}_{\Gamma,VB}|H'|\psi^{l,m}_{\Gamma,VB}\rangle e^{i(m-n)k_zc} && (1.4)\\
&\approx \frac{1}{N}\sum_n\left(\langle\psi^{u,n}_{\Gamma,VB}|H'|\psi^{l,n}_{\Gamma,VB}\rangle + \langle\psi^{u,n}_{\Gamma,VB}|H'|\psi^{l,n+1}_{\Gamma,VB}\rangle e^{ik_zc}\right) && (1.5)\\
&= \langle\psi^{u,n_0}_{\Gamma,VB}|H'|\psi^{l,n_0}_{\Gamma,VB}\rangle + \langle\psi^{u,n_0}_{\Gamma,VB}|H'|\psi^{l,n_0+1}_{\Gamma,VB}\rangle e^{ik_zc} && (1.6)\\
&\approx \frac{c_1^2}{2}\left(\langle p_z^{A,u,n_0}| - \langle p_z^{B,u,n_0}|\right)H'\left(|p_z^{A,l,n_0}\rangle - |p_z^{B,l,n_0}\rangle\right)\\
&\quad +\frac{c_1^2}{2}\left(\langle p_z^{A,u,n_0}| - \langle p_z^{B,u,n_0}|\right)H'\left(|p_z^{A,l,n_0+1}\rangle - |p_z^{B,l,n_0+1}\rangle\right)e^{ik_zc} && (1.7)\\
&\approx -\frac{c_1^2}{2}\langle p_z^{B,u,n_0}|H'|p_z^{A,l,n_0}\rangle - \frac{c_1^2}{2}\langle p_z^{A,u,n_0}|H'|p_z^{B,l,n_0+1}\rangle e^{ik_zc} && (1.8)
\end{aligned}$$

where we hold only the nearest neighboring hopping processes between p_z orbitals as leading orders in obtaining (1.5), (1.7) and (1.8). In (1.6), we apply the translational symmetry along z direction. The 2D Bloch wave functions are expressed as

$$|p_z^{A,l(u),n_0}\rangle = \frac{1}{\sqrt{N}_\parallel}\sum_{\mathbf{R}_\parallel^{A,l(u),n_0}}|p_z(\mathbf{R}_\parallel^{A,l(u),n_0})\rangle \tag{1.9}$$

and

$$|p_z^{B,l(u),n_0}\rangle = \frac{1}{\sqrt{N_\parallel}} \sum_{\mathbf{R}_\parallel^{B,l(u),n_0}} |p_z(\mathbf{R}_\parallel^{B,l(u),n_0})\rangle \tag{1.10}$$

where $\mathbf{R}_\parallel^{(A)B,l(u),n_0}$ represents the position vector of the X atom in the A(B) slab of the lower(upper) MX_2 layer in the n_0-th unit cell. $N_\parallel$ is the total number of X atoms in each slab. Note that there are no Bloch phase factors at Γ point. Then, the band splitting becomes

$$\begin{aligned}
\Delta_{\Gamma,VB}(k_z) &= -\frac{c_1^2}{2}\frac{1}{N_\parallel} \sum_{\mathbf{R}_\parallel^{B,u,n_0},\mathbf{R}_\parallel^{A,l,n_0}} \langle p_z(\mathbf{R}_\parallel^{B,u,n_0})|H'|p_z(\mathbf{R}_\parallel^{A,l,n_0})\rangle \\
&\quad -\frac{c_1^2}{2}\frac{1}{N_\parallel} \sum_{\mathbf{R}_\parallel^{A,u,n_0},\mathbf{R}_\parallel^{B,l,n_0+1}} \langle p_z(\mathbf{R}_\parallel^{A,u,n_0})|H'|p_z(\mathbf{R}_\parallel^{B,l,n_0+1})\rangle e^{ik_zc} && (1.11) \\
&= -\frac{c_1^2}{2}\frac{1}{N_\parallel} \sum_{\mathbf{R}_\parallel^{B,u,n_0}} \sum_i \langle p_z(\mathbf{R}_\parallel^{B,u,n_0})|H'|p_z(\mathbf{R}_\parallel^{B,u,n_0}+\boldsymbol{\delta}_i)\rangle && (1.12) \\
&\quad -\frac{c_1^2}{2}\frac{1}{N_\parallel} \sum_{\mathbf{R}_\parallel^{A,u,n_0}} \sum_i \langle p_z(\mathbf{R}_\parallel^{A,u,n_0})|H'|p_z(\mathbf{R}_\parallel^{A,u,n_0}+\tilde{\boldsymbol{\delta}}_i)\rangle e^{ik_zc} && (1.13) \\
&= -\frac{c_1^2}{2}\sum_i \langle p_z(\mathbf{R}_{\parallel,0}^{B,u,n_0})|H'|p_z(\mathbf{R}_{\parallel,0}^{B,u,n_0}+\boldsymbol{\delta}_i)\rangle - \frac{c_1^2}{2}\sum_i \langle p_z(\mathbf{R}_{\parallel,0}^{A,u,n_0})|H'|p_z(\mathbf{R}_{\parallel,0}^{A,u,n_0}+\tilde{\boldsymbol{\delta}}_i)\rangle e^{ik_zc} && (1.14)
\end{aligned}$$

where $\boldsymbol{\delta}_i$ represents three nearest neighboring sites between two planes in the same unit cell and $\tilde{\boldsymbol{\delta}}_i = -\boldsymbol{\delta}_i$ is for another pair of planes in different unit cells.[2] We make arbitrary choices, $\mathbf{R}_{\parallel,0}^{B,u,n_0}$ and $\mathbf{R}_{\parallel,0}^{A,u,n_0}$, for $\mathbf{R}_\parallel^{B,u,n_0}$ and $\mathbf{R}_\parallel^{A,u,n_0}$ regarding the translational symmetry.

For the evaluations of the overlap integrals, we exploit the Slater-Koster approximation for the p_z orbitals in different layers.:

$$\begin{aligned}
t_{p_i',p_j}^{(LL)}(\mathbf{r}_2-\mathbf{r}_1) &= \langle p_i'(\mathbf{r}_2)|H'|p_j'(\mathbf{r}_1)\rangle && (1.15) \\
&= (V_{pp\sigma}-V_{pp\pi})\frac{r_ir_j}{r^2} + V_{pp\pi}\delta_{ij} && (1.16)
\end{aligned}$$

where $\mathbf{r} = \mathbf{r}_2 - \mathbf{r}_1$.[1] Since only p_z orbitals are involved in calculating $\Delta_{\Gamma,VB}(k_z)$, we have $r_ir_j/r^2 = (\delta_z/\delta)^2$. Here, δ and δ_z are distances between X atoms belonging to the nearest layers as illustrated in Fig. 2. Noticing that all the three nearest neighbors $\boldsymbol{\delta}_i$ have the same magnitude of their z component, the band splitting reduces to

$$\Delta_{\Gamma,VB}(k_z) = -\frac{D_\Gamma}{2}\left(1+e^{ik_zc}\right) \tag{1.17}$$

where

$$D_\Gamma = 3c_1^2\left\{(V_{pp\sigma}-V_{pp\pi})\left(\frac{\delta_z}{\delta}\right)^2 + V_{pp\pi}\right\}. \tag{1.18}$$

Then, the energy spectra along k_z direction at the in-plane Γ point is given by

$$E_{\Gamma,VB}^{\pm}(k_z) \approx \epsilon_{\Gamma,VB} \pm D_\Gamma \cos\frac{k_z}{2}. \tag{1.19}$$

II. k_z DISPERSION AT THE IN-PLANE K POINT

In this section, we deal with the conduction and valence spectra along $\mathbf{K} + k_z\hat{z}$. Since two bands of the monolayer MX_2 are involved, we have four basis Bloch wave functions of the bulk system given by

$$|\Psi_{K,VB}^{l(u)}(k_z)\rangle = \frac{1}{\sqrt{N}}\sum_n |\psi_{K,VB}^{l(u),n}\rangle e^{ink_zc} \quad \text{and} \quad |\Psi_{K,CB}^{l(u)}(k_z)\rangle = \frac{1}{\sqrt{N}}\sum_n |\psi_{K,CB}^{l(u),n}\rangle e^{ink_zc} \tag{2.1}$$

where

$$|\psi^{l(u),n}_{K,VB}\rangle = \tilde{c}_6|d^{(e)}_2\rangle + c_6|p^{(e)}_1\rangle \quad \text{and} \quad |\psi^{l(u),n}_{K,CB}\rangle = \tilde{c}_5|d^{(e)}_0\rangle + c_5|p^{(e)}_{-1}\rangle. \tag{2.2}$$

Here, $|d^{(e)}_0 = |d_{z^2}\rangle$, $|d^{(e)}_2\rangle = (|d^{(e)}_{x^2-y^2}\rangle + i|d^{(e)}_{xy}\rangle)/\sqrt{2}$ and $|p^{(e)}_{\pm1}\rangle = \mp\{(|p^A_x\rangle + |p^B_x\rangle) \pm i(|p^A_y\rangle + |p^B_y\rangle)\}/2.$[1]

With the basis set $\{|\Psi^u_{K,VB}(k_z)\rangle, |\Psi^u_{K,CB}(k_z)\rangle, |\Psi^l_{K,VB}(k_z)\rangle, |\Psi^l_{K,CB}(k_z)\rangle\}$ we obtain the effective 4 Hamiltonian expressed as

$$H_K(k_z) = \begin{pmatrix} \epsilon_{K,VB} & 0 & \Delta_{K,VB}(k_z) & \alpha_K(k_z) \\ 0 & \epsilon_{K,CB} & \beta_K(k_z) & \Delta_{K,CB}(k_z) \\ \Delta^*_{K,VB}(k_z) & \beta^*_K(k_z) & \epsilon_{K,VB} & 0 \\ \alpha^*_K(k_z) & \Delta^*_{K,CB}(k_z) & 0 & \epsilon_{K,CB} \end{pmatrix} \tag{2.3}$$

where $\epsilon_{K,VB}$ and $\epsilon_{K,CB}$ are the on-site energy of the valence and conduction bands of the MX_2 monolayer at K point.

The off-diagonal elements are calculated as follows. First, let us consider $\Delta_{K,VB}(k_z)$ which is the coupling valence electrons in the nearest neighboring MX_2 layers.

$$\begin{aligned}
\Delta_{K,VB}(k_z) &= \langle\Psi^u_{K,VB}(k_z)|H'|\Psi^l_{K,VB}(k_z)\rangle && (2.4)\\
&= \frac{1}{N}\sum_{n,m}\langle\psi^{u,n}_{K,VB}|H'|\psi^{l,m}_{K,VB}\rangle e^{i(m-n)k_z c} && (2.5)\\
&\approx \frac{1}{N}\sum_{n}\Big(\langle\psi^{u,n}_{K,VB}|H'|\psi^{l,n}_{K,VB}\rangle + \langle\psi^{u,n}_{K,VB}|H'|\psi^{l,n+1}_{K,VB}\rangle e^{ik_z c}\Big) && (2.6)\\
&= \langle\psi^{u,n_0}_{K,VB}|H'|\psi^{l,n_0}_{K,VB}\rangle + \langle\psi^{u,n_0}_{K,VB}|H'|\psi^{l,n_0+1}_{K,VB}\rangle e^{ik_z c} && (2.7)\\
&\approx \frac{c_6^2}{4}\big\{-\langle p^{A,u,n_0}_x| - \langle p^{B,u,n_0}_x| + i\left(\langle p^{A,u,n_0}_y| + \langle p^{B,u,n_0}_y|\right)\big\}H'\big\{-|p^{A,l,n_0}_x\rangle - |p^{B,l,n_0}_x\rangle \\
&\quad -i\left(|p^{A,l,n_0}_y\rangle + |p^{B,l,n_0}_y\rangle\right)\big\} + \frac{c_6^2}{4}\big\{-\langle p^{A,u,n_0}_x| - \langle p^{B,u,n_0}_x| + i\left(\langle p^{A,u,n_0}_y| + \langle p^{B,u,n_0}_y|\right)\big\}H' \\
&\quad \times\big\{-|p^{A,l,n_0+1}_x\rangle - |p^{B,l,n_0+1}_x\rangle - i\left(|p^{A,l,n_0+1}_y\rangle + |p^{B,l,n_0+1}_y\rangle\right)\big\}e^{ik_z c} && (2.8)\\
&\approx \frac{c_6^2}{4}\big\{\langle p^{B,u,n_0}_x| - i\langle p^{B,u,n_0}_y|\big\}H'\big\{|p^{A,l,n_0}_x\rangle + i|p^{A,l,n_0}_y\rangle\big\} \\
&\quad +\frac{c_6^2}{4}\big\{\langle p^{A,u,n_0}_x| - i\langle p^{A,u,n_0}_y|\big\}H'\big\{|p^{B,l,n_0+1}_x\rangle + i|p^{B,l,n_0+1}_y\rangle\big\}e^{ik_z c} && (2.9)
\end{aligned}$$

where all the approximations are made by taking the leading terms. Again, those bras and kets are the Bloch states of following forms.:

$$|p^{A,l(u),n_0}_{x(y)}\rangle = \frac{1}{\sqrt{N}_\parallel}\sum_{\mathbf{R}^{A,l(u),n_0}_\parallel}|p_{x(y)}(\mathbf{R}^{A,l(u),n_0}_\parallel)\rangle e^{i\mathbf{K}\cdot\mathbf{R}^{A,l(u),n_0}_\parallel} \tag{2.10}$$

and

$$|p^{B,l(u),n_0}_{x(y)}\rangle = \frac{1}{\sqrt{N}_\parallel}\sum_{\mathbf{R}^{B,l(u),n_0}_\parallel}|p_{x(y)}(\mathbf{R}^{B,l(u),n_0}_\parallel)\rangle e^{i\mathbf{K}\cdot\mathbf{R}^{B,l(u),n_0}_\parallel}. \tag{2.11}$$

Then, $\Delta_{K,VB}(k_z)$ becomes

$$\begin{aligned}
\Delta_{K,VB}(k_z) &= \frac{c_6^2}{4}\frac{1}{N_\parallel}\sum_{\mathbf{R}_\parallel^{B,u,n_0},\mathbf{R}_\parallel^{A,l,n_0}}\Bigg[\left\{\langle p_x(\mathbf{R}_\parallel^{B,u,n_0})| - i\langle p_y(\mathbf{R}_\parallel^{B,u,n_0})|\right\} H' \left\{|p_x(\mathbf{R}_\parallel^{A,l,n_0})\rangle + i|p_y(\mathbf{R}_\parallel^{A,l,n_0})\rangle\right\} \\
&\quad \times e^{i\mathbf{K}\cdot\left(\mathbf{R}_\parallel^{A,l,n_0}-\mathbf{R}_\parallel^{B,u,n_0}\right)}\Bigg] + \frac{c_6^2}{4}\frac{1}{N_\parallel}\sum_{\mathbf{R}_\parallel^{A,u,n_0},\mathbf{R}_\parallel^{B,l,n_0+1}}\Bigg[\left\{\langle p_x(\mathbf{R}_\parallel^{A,u,n_0})| - i\langle p_y(\mathbf{R}_\parallel^{A,u,n_0})|\right\} H' \\
&\quad \times\left\{|p_x(\mathbf{R}_\parallel^{B,l,n_0+1})\rangle + i|p_y(\mathbf{R}_\parallel^{B,l,n_0+1})\rangle\right\} e^{i\mathbf{K}\cdot\left(\mathbf{R}_\parallel^{B,l,n_0+1}-\mathbf{R}_\parallel^{A,u,n_0}\right)}\Bigg]e^{ik_zc} \qquad (2.12) \\
&= \frac{c_6^2}{4}\sum_i\left\{\langle p_x(\mathbf{R}_{\parallel,0}^{B,u,n_0})| - i\langle p_y(\mathbf{R}_{\parallel,0}^{B,u,n_0})|\right\} H'\left\{|p_x(\mathbf{R}_{\parallel,0}^{B,u,n_0}+\boldsymbol{\delta}_i)\rangle + i|p_y(\mathbf{R}_{\parallel,0}^{B,u,n_0}+\boldsymbol{\delta}_i))\rangle\right\}e^{i\mathbf{K}\cdot\boldsymbol{\delta}_i} \\
&\quad + \frac{c_6^2}{4}\sum_i\Bigg[\left\{\langle p_x(\mathbf{R}_{\parallel,0}^{A,u,n_0})| - i\langle p_y(\mathbf{R}_{\parallel,0}^{A,u,n_0})|\right\} H'\left\{|p_x(\mathbf{R}_{\parallel,0}^{A,u,n_0}+\tilde{\boldsymbol{\delta}}_i)\rangle + i|p_y(\mathbf{R}_{\parallel,0}^{A,u,n_0}+\tilde{\boldsymbol{\delta}}_i))\rangle\right\} \\
&\quad \times e^{i\mathbf{K}\cdot\tilde{\boldsymbol{\delta}}_i}\Bigg]e^{ik_zc} \qquad (2.13) \\
&= \frac{c_6^2}{4}\sum_i\left\{\langle p_x(\mathbf{R}_{\parallel,0}^{B,u,n_0})|H'|p_x(\mathbf{R}_{\parallel,0}^{B,u,n_0}+\boldsymbol{\delta}_i)\rangle + \langle p_y(\mathbf{R}_{\parallel,0}^{B,u,n_0})|H'|p_y(\mathbf{R}_{\parallel,0}^{B,u,n_0}+\boldsymbol{\delta}_i)\rangle\right\}e^{i\mathbf{K}\cdot\boldsymbol{\delta}_i} \\
&\quad + \frac{c_6^2}{4}\sum_i\left\{\langle p_x(\mathbf{R}_{\parallel,0}^{A,u,n_0})|H'|p_x(\mathbf{R}_{\parallel,0}^{A,u,n_0}+\tilde{\boldsymbol{\delta}}_i)\rangle + \langle p_y(\mathbf{R}_{\parallel,0}^{A,u,n_0})|H'|p_y(\mathbf{R}_{\parallel,0}^{A,u,n_0}+\tilde{\boldsymbol{\delta}}_i)\rangle\right\}e^{i\mathbf{K}\cdot\tilde{\boldsymbol{\delta}}_i}e^{ik_zc} \qquad (2.14)
\end{aligned}$$

where we apply the fact that $t^{(LL)}_{p_i',p_j}$ is invariant under $i \leftrightarrow j$ so that the terms involving p_x and p_y simultaneously cancel each other. Then, using the Slater-Koster formula, we have

$$\begin{aligned}
\Delta_{K,VB}(k_z) &= \frac{c_6^2}{4}\sum_i\left\{(V_{pp\sigma}-V_{pp\pi})\frac{\delta_{i,\parallel}^2}{\delta^2}+2V_{pp\pi}\right\}e^{i\mathbf{K}\cdot\boldsymbol{\delta}_i} + \frac{c_6^2}{4}\sum_i\left\{(V_{pp\sigma}-V_{pp\pi})\frac{\tilde{\delta}_{i,\parallel}^2}{\tilde{\delta}^2}+2V_{pp\pi}\right\}e^{i\mathbf{K}\cdot\tilde{\boldsymbol{\delta}}_i}e^{ik_zc} \qquad (2.15) \\
&= \frac{c_6^2}{4}\sum_i\left\{(V_{pp\sigma}-V_{pp\pi})\frac{\delta_{i,\parallel}^2}{\delta^2}+2V_{pp\pi}\right\}\left(e^{i\mathbf{K}\cdot\boldsymbol{\delta}_i}+e^{i\mathbf{K}\cdot\tilde{\boldsymbol{\delta}}_i}e^{ik_zc}\right) \qquad (2.16) \\
&= \frac{c_6^2}{4}\left\{(V_{pp\sigma}-V_{pp\pi})\frac{\delta_\parallel^2}{\delta^2}+2V_{pp\pi}\right\}\sum_i\left(e^{i\mathbf{K}\cdot\boldsymbol{\delta}_i}+e^{-i\mathbf{K}\cdot\boldsymbol{\delta}_i}e^{ik_zc}\right) \qquad (2.17)
\end{aligned}$$

where we use the fact that $\delta_\parallel^2 \equiv \delta_{i,\parallel}^2 = \delta_{i,x}^2+\delta_{i,y}^2$ is independent of i due to the C_3 symmetry. With $\delta_{1,\parallel} = (a/2, a/2\sqrt{3}, 0)$, $\delta_{2,\parallel} = (-a/2, a/2\sqrt{3}, 0)$ and $\delta_{3,\parallel} = (0, -a/\sqrt{3}, 0)$, one can show that $\sum_i e^{i\mathbf{K}\cdot\boldsymbol{\delta}_i} = \sum_i e^{-i\mathbf{K}\cdot\boldsymbol{\delta}_i} = 0$ which leads to $\Delta_{K,VB}(k_z) = 0$. Similarly, one can also show that $\Delta_{K,CB}(k_z) = 0$.

Finally, we evaluate $\alpha_K(k_z)$ and $\beta_K(k_z)$ which are overlap integrals between electrons in the valence and conduction

bands.

$$\begin{aligned}
\alpha_K(k_z) &= \langle \Psi^u_{K,VB}(k_z)|H'|\Psi^l_{K,CB}(k_z)\rangle && (2.18)\\
&= \frac{1}{N}\sum_{n,m}\langle \psi^{u,n}_{K,VB}|H'|\psi^{l,m}_{K,CB}\rangle e^{i(m-n)k_zc} && (2.19)\\
&\approx \frac{1}{N}\sum_{n}\Big(\langle \psi^{u,n}_{K,VB}|H'|\psi^{l,n}_{K,CB}\rangle + \langle \psi^{u,n}_{K,VB}|H'|\psi^{l,n+1}_{K,CB}\rangle e^{ik_zc}\Big) && (2.20)\\
&= \langle \psi^{u,n_0}_{K,VB}|H'|\psi^{l,n_0}_{K,CB}\rangle + \langle \psi^{u,n_0}_{K,VB}|H'|\psi^{l,n_0+1}_{K,CB}\rangle e^{ik_zc} && (2.21)\\
&\approx \frac{c_6c_5}{4}\big\{-\langle p_x^{A,u,n_0}| - \langle p_x^{B,u,n_0}| + i\left(\langle p_y^{A,u,n_0}| + \langle p_y^{B,u,n_0}|\right)\big\}H'\big\{|p_x^{A,l,n_0}\rangle + |p_x^{B,l,n_0}\rangle\\
&\quad -i\left(|p_y^{A,l,n_0}\rangle + |p_y^{B,l,n_0}\rangle\right)\big\} + \frac{c_6c_5}{4}\big\{-\langle p_x^{A,u,n_0}| - \langle p_x^{B,u,n_0}| + i\left(\langle p_y^{A,u,n_0}| + \langle p_y^{B,u,n_0}|\right)\big\}H'\\
&\quad \times\big\{|p_x^{A,l,n_0+1}\rangle + |p_x^{B,l,n_0+1}\rangle - i\left(|p_y^{A,l,n_0+1}\rangle + |p_y^{B,l,n_0+1}\rangle\right)\big\}e^{ik_zc} && (2.22)\\
&\approx -\frac{c_6c_5}{4}\big\{\langle p_x^{B,u,n_0}| - i\langle p_y^{B,u,n_0}|\big\}H'\big\{|p_x^{A,l,n_0}\rangle - i|p_y^{A,l,n_0}\rangle\big\}\\
&\quad -\frac{c_6c_5}{4}\big\{\langle p_x^{A,u,n_0}| - i\langle p_y^{A,u,n_0}|\big\}H'\big\{|p_x^{B,l,n_0+1}\rangle - i|p_y^{B,l,n_0+1}\rangle\big\}e^{ik_zc} && (2.23)\\
&= -\frac{c_6c_5}{4}\sum_i\left\{\langle p_x(\mathbf{R}^{B,u,n_0}_{\|,0})| - i\langle p_y(\mathbf{R}^{B,u,n_0}_{\|,0})|\right\}H'\left\{|p_x(\mathbf{R}^{B,u,n_0}_{\|,0}+\boldsymbol{\delta}_i)\rangle - i|p_y(\mathbf{R}^{B,u,n_0}_{\|,0}+\boldsymbol{\delta}_i))\rangle\right\}e^{i\mathbf{K}\cdot\boldsymbol{\delta}_i}\\
&\quad -\frac{c_6c_5}{4}\sum_i\Bigg[\left\{\langle p_x(\mathbf{R}^{A,u,n_0}_{\|,0})| - i\langle p_y(\mathbf{R}^{A,u,n_0}_{\|,0})|\right\}H'\left\{|p_x(\mathbf{R}^{A,u,n_0}_{\|,0}+\tilde{\boldsymbol{\delta}}_i)\rangle - i|p_y(\mathbf{R}^{A,u,n_0}_{\|,0}+\tilde{\boldsymbol{\delta}}_i))\rangle\right\}\\
&\quad \times e^{i\mathbf{K}\cdot\tilde{\boldsymbol{\delta}}_i}\Bigg]e^{ik_zc} && (2.24)\\
&= -\frac{c_6c_5}{4}\sum_i\Big\{\langle p_x(\mathbf{R}^{B,u,n_0}_{\|,0})|H'|p_x(\mathbf{R}^{B,u,n_0}_{\|,0}+\boldsymbol{\delta}_i)\rangle - \langle p_y(\mathbf{R}^{B,u,n_0}_{\|,0})|H'|p_y(\mathbf{R}^{B,u,n_0}_{\|,0}+\boldsymbol{\delta}_i)\rangle\\
&\quad -2i\langle p_x(\mathbf{R}^{B,u,n_0}_{\|,0})|H'|p_y(\mathbf{R}^{B,u,n_0}_{\|,0}+\boldsymbol{\delta}_i)\rangle\Big\}e^{i\mathbf{K}\cdot\boldsymbol{\delta}_i} - \frac{c_6c_5}{4}\sum_i\Big\{\langle p_x(\mathbf{R}^{A,u,n_0}_{\|,0})|H'|p_x(\mathbf{R}^{A,u,n_0}_{\|,0}+\tilde{\boldsymbol{\delta}}_i)\rangle\\
&\quad -\langle p_y(\mathbf{R}^{A,u,n_0}_{\|,0})|H'|p_y(\mathbf{R}^{A,u,n_0}_{\|,0}+\tilde{\boldsymbol{\delta}}_i)\rangle - 2i\langle p_x(\mathbf{R}^{A,u,n_0}_{\|,0})|H'|p_y(\mathbf{R}^{A,u,n_0}_{\|,0}+\tilde{\boldsymbol{\delta}}_i)\rangle\Big\}e^{i\mathbf{K}\cdot\tilde{\boldsymbol{\delta}}_i}e^{ik_zc}. && (2.25)
\end{aligned}$$

If we apply the Slater-Koster approximation, it reduces to

$$\begin{aligned}
\alpha_K(k_z) &= -\frac{c_5c_6}{4}\sum_i\left\{(V_{pp\sigma}-V_{pp\pi})\frac{(\delta_{i,x}-i\delta_{i,y})^2}{\delta_i^2} + 2V_{pp\pi}\right\}e^{i\mathbf{K}\cdot\boldsymbol{\delta}_i}\\
&\quad -\frac{c_5c_6}{4}\sum_i\left\{(V_{pp\sigma}-V_{pp\pi})\frac{(\tilde{\delta}_{i,x}-i\tilde{\delta}_{i,y})^2}{\tilde{\delta}_i^2} + 2V_{pp\pi}\right\}e^{i\mathbf{K}\cdot\tilde{\boldsymbol{\delta}}_i}e^{ik_zc} && (2.26)\\
&= -\frac{c_5c_6}{4}(V_{pp\sigma}-V_{pp\pi})\left(\frac{\delta_\|}{\delta}\right)^2\sum_i\left\{\frac{(\delta_{i,x}-i\delta_{i,y})^2}{\delta_\|^2}e^{i\mathbf{K}\cdot\boldsymbol{\delta}_i} + \frac{(\tilde{\delta}_{i,x}-i\tilde{\delta}_{i,y})^2}{\tilde{\delta}_\|^2}e^{i\mathbf{K}\cdot\tilde{\boldsymbol{\delta}}_i}e^{ik_zc}\right\} && (2.27)\\
&= \frac{3c_5c_6}{4}(V_{pp\sigma}-V_{pp\pi})\left(\frac{\delta_\|}{\delta}\right)^2 e^{ik_zc} && (2.28)\\
&= D_K e^{ik_zc} && (2.29)
\end{aligned}$$

Similarly, we obtain

$$\begin{aligned}
\beta_K(k_z) &= \langle \Psi^u_{K,CB}(k_z)|H'|\Psi^l_{K,VB}(k_z)\rangle && (2.30)\\
&\approx -\frac{c_5c_6}{4}(V_{pp\sigma}-V_{pp\pi})\left(\frac{\delta_\|}{\delta}\right)^2\sum_i\left\{\frac{(\delta_{i,x}+i\delta_{i,y})^2}{\delta_\|^2}e^{i\mathbf{K}\cdot\boldsymbol{\delta}_i} + \frac{(\tilde{\delta}_{i,x}+i\tilde{\delta}_{i,y})^2}{\tilde{\delta}_\|^2}e^{i\mathbf{K}\cdot\tilde{\boldsymbol{\delta}}_i}e^{ik_zc}\right\} && (2.31)\\
&= \frac{3c_5c_6}{4}(V_{pp\sigma}-V_{pp\pi})\left(\frac{\delta_\|}{\delta}\right)^2 && (2.32)\\
&= D_K. && (2.33)
\end{aligned}$$

One can check we arrive at the same result at K′ point.

With above results, the effective Hamiltonian becomes

$$H_K \approx \begin{pmatrix} \epsilon_{K,VB} & 0 & 0 & D_K e^{ik_z c} \\ 0 & \epsilon_{K,CB} & D_K & 0 \\ 0 & D_K & \epsilon_{K,VB} & 0 \\ D_K e^{-ik_z c} & 0 & 0 & \epsilon_{K,CB} \end{pmatrix} \tag{2.34}$$

whose eigenvalues are evaluated as

$$E_K^{\pm} = \frac{\epsilon_{K,VB} + \epsilon_{K,CB} \pm \sqrt{(\epsilon_{K,VB} - \epsilon_{K,CB})^2 + 4D_K^2}}{2} \tag{2.35}$$

which is independent of k_z. For $|\epsilon_{K,VB} - \epsilon_{K,CB}| \gg D_K$, the energy spectra of the valence and conduction bands change as

$$\epsilon_{K,VB} \to \epsilon_{K,VB} - \frac{D_K^2}{\epsilon_{K,CB} - \epsilon_{K,VB}} \tag{2.36}$$

and

$$\epsilon_{K,CB} \to \epsilon_{K,CB} + \frac{D_K^2}{\epsilon_{K,CB} - \epsilon_{K,VB}}. \tag{2.37}$$

---

* Electronic address: phyruth@gmail.com
† Electronic address: AbePark@incheon.ac.kr

1 S. Fang, R. KuateDefo, S. N. Shirodkar, S. Lieu, G. A. Tritsaris, and E. Kaxiras, Phys. Rev. B **92**, 205108 (2015).
2 X. Su, R. Z. Zhang, C. F. Guo, M. Guoc, and Z. Y. Rena, Phys. Chem. Chem. Phys. **16** 1393 (2014).
